# Numerical Simulations as Means for Tailoring Electrically Conductive Hydrogels towards Cartilage Tissue Engineering by Electrical Stimulation

**DOI:** 10.3390/molecules25204750

**Published:** 2020-10-16

**Authors:** Julius Zimmermann, Thomas Distler, Aldo R. Boccaccini, Ursula van Rienen

**Affiliations:** 1 Institute of General Electrical Engineering, University of Rostock, 18051 Rostock, Germany; ursula.van-rienen@uni-rostock.de; 2 Institute of Biomaterials, Friedrich Alexander University Erlangen-Nuremberg, 91058 Erlangen, Germany; thomas.distler@fau.de (T.D.); aldo.boccaccini@fau.de (A.R.B.); 3 Department Life, Light & Matter, University of Rostock, 18051 Rostock, Germany; 4 Department of Ageing of Individuals and Society, Interdisciplinary Faculty, University of Rostock, 18051 Rostock, Germany

**Keywords:** electrical stimulation, electrically conductive hydrogels, tissue engineering, scaffold, computational modelling, uncertainty quantification, capacitive coupling, finite element analysis, biomaterial scaffolds

## Abstract

Cartilage regeneration is a clinical challenge. In recent years, hydrogels have emerged as implantable scaffolds in cartilage tissue engineering. Similarly, electrical stimulation has been employed to improve matrix synthesis of cartilage cells, and thus to foster engineering and regeneration of cartilage tissue. The combination of hydrogels and electrical stimulation may pave the way for new clinical treatment of cartilage lesions. To find the optimal electric properties of hydrogels, theoretical considerations and corresponding numerical simulations are needed to identify well-suited initial parameters for experimental studies. We present the theoretical analysis of a hydrogel in a frequently used electrical stimulation device for cartilage regeneration and tissue engineering. By means of equivalent circuits, finite element analysis, and uncertainty quantification, we elucidate the influence of the geometric and dielectric properties of cell-seeded hydrogels on the capacitive-coupling electrical field stimulation. Moreover, we discuss the possibility of cellular organisation inside the hydrogel due to forces generated by the external electric field. The introduced methodology is easily reusable by other researchers and allows to directly develop novel electrical stimulation study designs. Thus, this study paves the way for the design of future experimental studies using electrically conductive hydrogels and electrical stimulation for tissue engineering.

## 1. Introduction

Severe articular cartilage lesions are painful and may eventually make an artificial joint inevitable. To prevent this, regenerative therapies to treat articular cartilage lesions and related diseases such as osteoarthritis are needed. To date, different clinical approaches to repair or regenerate articular cartilage have been established [1,2]. Among them, scaffold-based techniques using hydrogels are an emerging and promising field [2,3,4,5]. Hydrogels are particularly attractive as they can resemble the native extracellular matrix (ECM) of tissues. As they can be tuned for 3D printability, they show promise as a strategy to engineer patient-specific solutions to develop novel cartilage tissue repair implants [6,7].

Articular cartilage represents one type of cartilage, the so-called hyaline cartilage. However, fibrocartilage often forms during its regeneration process [1,2]. Its mechanical properties are disadvantageous in comparison to the physiological hyaline cartilage. This unwanted behaviour is indicated by an increase of collagen type I and a simultaneous decrease of collagen type II as well as a decrease of glycosaminoglycans content in the ECM [1,8,9]. It stems from a de-differentiation of the cartilage cells, the chondrocytes [8,9]. Electrical stimulation (ES) has been identified as a cue to remedy the de-differentiation of chondrocytes by supporting re-differentiation [9,10] or enhance chondrogenic differentiation of stem cells [11]. Moreover, ES has been shown to increase ECM synthesis [12,13,14].

**Figure 1:**
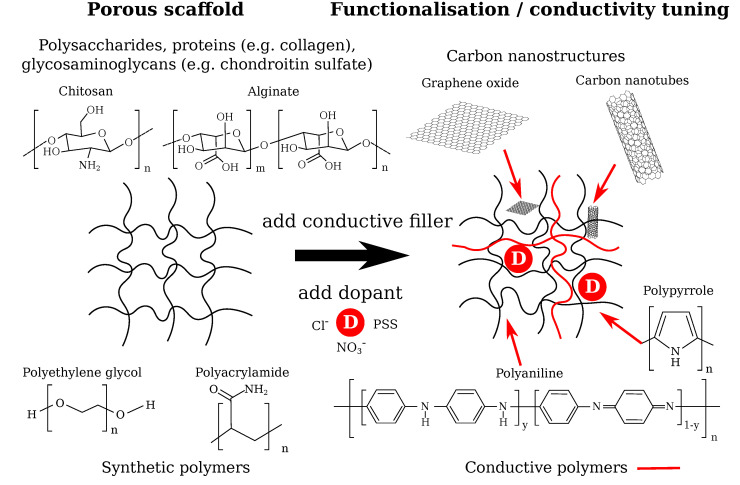
Potential design routes for electrically conductive scaffolds to be used in cartilage tissue engineering. Possible materials for the porous, non-conductive scaffold and for the conductivity tuning using conductive fillers and dopants are shown. The dopant changes the conductivity of the scaffold by adding or removing an electron from/to the polymer, which causes a lattice distortion inducing polarons that yield increased electric conductivity [19]. Carbon nanostructures can be integrated into the scaffold network and provide a pathway for the electric current [20,21]. The final electroactive hydrogel conductivity will be strongly dependent on the degree of percolation between the conductive fillers, purity and crystalinity of the conductive polymer, doping level, redox state of the conductive filler, diffusibility of and ion mobility in the final hydrogel, hydrogel porosity, and additional factors relevant for tuning the final hydrogel conductivity.

To enhance the outcome of ES on the cellular response, electroactive hydrogels represent a promising potential strategy. The design of such hydrogels (Figure 1) comprises the choice of the scaffold material and conductivity tuning by appropriate electroactive materials [15,16] or by initially using hydrogels solely consisting of conductive polymers [17]. Such conductive polymer composites have been shown to be suitable for biomedical applications [15]. Biomaterials that served as scaffolds for cartilage tissue engineering (CTE) exemplarily included natural polysaccharides (alginate, agarose, chitosan) or constituents of the ECM (collagen, chondroitin sulfate, hyaluronic acid) as well as synthetic polymers (e.g., polyethylene glycol, polyacrylamide) [18]. Improved functionality can be reached by adding conductive polymers (polypyrrole, polyaniline) [16,19] or carbon nanostructures (graphene oxide, carbon nanotubes) [20] capable of enhancing the intrinsic hydrogel conductivity. When using conductive polymers, suitable dopants—for example, small salt ions (Cl^−^, NO_3_^−^) or large polyelectrolyte molecules (e.g., sodium polystyrenesulfonate (PSS))—are a key ingredient for adjusting the conductivity of the hydrogel [15,19].

However, different constraints arise due to the function of articular cartilage. The mechanical properties of the hydrogel should match the properties of native articular cartilage [18] or be adapted to allow for optimized ECM production by, e.g., fast stress relaxation or lower stiffness [22]. Furthermore, it has been shown that negatively charged hydrogels have superior cell attachment and support expression of ECM proteins in comparison to positively charged hydrogels [23]. Functionalised hydrogels fulfilling these criteria (hence they are suitable for CTE) have been developed [21,24,25]. Synthetic polymers augmented with graphene oxide (GO) with tuneable conductivity have successfully been tested in vivo [21,24]. On the other hand, an oligopyrrole/chitosan hydrogel doped with NaClO_4_ has been suggested for CTE [25]. To fabricate hydrogels that mimic the complexity of articular cartilage, 3D printing techniques have been identified as a promising route [26,27]. The interaction of the scaffolds with the chondrocytes depends on the structure of the scaffold. For example, in the case of fibrous collagen scaffolds, the scaffold fibres should have a smaller diameter than the cells to ensure a spherical cell shape [28] that is beneficial for chondrogenesis [29]. Spherical chondrocytes have occured dominantly in sponge-like collagen scaffolds [28]. Printed scaffolds allow to include tailored macroscopic porosity in the range of several hundred microns as well as microporosity by the hydrogel itself. In addition, hydrogel porosity can be tuned by, e.g., freeze-drying techniques to optimize the microporosity towards increased cell–material interaction [30,31]. Furthermore, recent research has demonstrated the 3D printing of electroactive hydrogels with tuneable conductivity [7,27]. Both the conductivity of about $1.5\;{S\;m}^{-1}$, which is commonly assumed for a cell culture medium [32], and $1\;{S\;m}^{-1}$, which is approximately the conductivity of bovine articular cartilage [33], are included in the feasible range. Hence, established 3D printing techniques can be employed to manufacture hydrogels that mimic the electrical properties of the cellular ECM environment and of common in vitro cell culture conditions. Unlike the conductivity, the permittivity of the scaffolds has usually not been investigated and it is not clear if it can be tailored as well.

In this study, we focus on capacitive coupling ES as it has been widely tested in vitro for CTE and cartilage regeneration [9,11,12,13,34,35,36,37,38,39]. Typically, frequencies in the kHz range have been used, with 60 kHz as the most popular choice. The stimulating electric field usually has had a field strength of $2\;{V\;m}^{-1}$ [12,13,35,36,37,38]. Recently, smaller field strengths of $1\;{V\;m}^{-1}$ [11] and about $5\;\mu {V\;m}^{-1}$ have also been considered [9]. The capacitively coupled stimulation has been applied on cell cultures [9,12,34,35,37,39] and cartilage explants [13,36,38]. Other potential strategies of ES for CTE could be based on direct contact stimulation, where the electrode is in direct contact with the sample [40]. In this approach, electrochemical interactions between the electrode and the cell culture medium may occur. Agar salt bridges can reduce such interactions [41]. Even though the use of ES for CTE has been explored, still a clear explanation of the underlying mechanisms is lacking. Thus, there is need for further research given the current limitations to pave the way for regenerative therapies and stimulation devices based on ES for CTE.

The focus of this study is to describe a capacitive-coupling ES system for CTE [35,42] by theoretical means. We first review a standard experimental set-up that delivers the electric fields by capacitive coupling to the biological sample [35,42]. Using equivalent circuit analysis together with finite element analysis (FEA), a comprehensible and validated representation of the experimental set-up is found. Subsequently, we present our results of a numerical model, which sheds light onto the possible effect of the ES on the cells immersed in an electrically conductive hydrogel. A measure of the effect of the stimulation is the induced transmembrane potential (TMP) [40,43]. It is defined as the difference between the potential inside and outside the cell membrane. The change in the TMP leads to an opening of voltage-gated ion channels [37,40,44]. In the context of cartilage engineering, the positive effect of the discussed stimulation set-up has been linked to the activation of voltage-gated calcium channels [37]. To understand the influence of the hydrogel properties at different stages of the experiment, we analyse the induced TMP of a single cell for three different configurations: (1) an elongated, flat cell corresponding to initial cell adhesion to the hydrogel, (2) the transition to spherical cell shape (half grown-in), and (3) a fully embedded spherical cell, which is the desired shape supporting chondrogenesis [29]. The latter configuration corresponds to either an encapsulated cell shown in direct cell printing or to cells migrated into the hydrogel. As these models may be sensitive towards the choice of uncertain parameters, we combine our analysis with uncertainty quantification (UQ) to include the uncertainty of various parameters such as the dielectric properties of the hydrogel and the cell. We determined the importance of the individual parameters with regard to the effect of the ES and its experimental validation. In our FEA, we find that the hydrogel conductivity plays an important role for the stimulation effect if one considers the TMP as a marker for effective stimulation. A lower conductivity contributes to an increased TMP value. In contrast, the effect of the hydrogel permittivity on ES is more or less negligible. Significant differences between the cell shapes and locations also become evident. Finally, we conclude our study by investigating the possibility of a cellular organisation in the hydrogel or cell deformation due to a mechanical effect of the ES. We link the possible effect of the hydrogel conductivity to the stimulation outcome and find that high-conductivity scaffolds might support the mechanical effect if it exists. In sum, this study contributes to the development of electrically conductive scaffolds for cartilage tissue engineering.

## 2. Results and Discussion

### 2.1. Validation of the Modelling Approach

**Figure 2:**
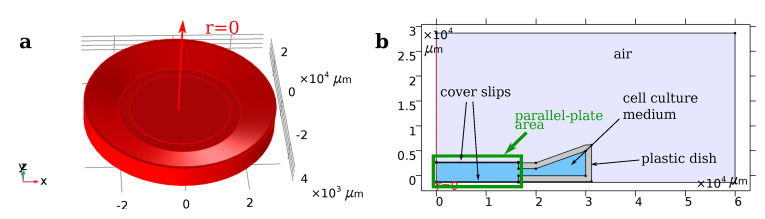
General geometry of the simulation model. (**a**) 3D representation of the axisymmetric set-up, consisting of (**b**): air, culture medium/buffer (blue), plastic dish (grey), and symmetry axis (red). The voltage is applied between the top side of the upper cover slip and the bottom side of the lower cover slip. The area highlighted in green is the area for which the parallel-plate capacitor approximation was applied.

The capacitive-coupling chamber (Figure 2) can be well described by an equivalent circuit comprising two capacitors and a lossy dielectric.

The two cover slips separating the electrode from the medium act as capacitors, whereas the cell culture medium is the lossy dielectric. Using the complex permittivity representation [45], the total impedance of the device is given by

(1) $$Z_{\mathrm{total}}=2Z_{\mathrm{cs}}+Z_{\mathrm{buf}}=\frac{2d_{\mathrm{cs}}}{i\omega \varepsilon_{\mathrm{cs}}^{*}\pi r_{\mathrm{cs}}^{2}}+\frac{d_{\mathrm{buf}}}{i\omega \varepsilon_{\mathrm{buf}}^{*}\pi r_{\mathrm{buf}}^{2}},$$

where $\varepsilon^{*}$ is the complex permittivity, *d* is the thickness, and *r* the radius of the respective part of the geometry. The respective properties of the cover slips and medium are denoted by subscripts *cs* and *buf*. The electric field in the cell culture medium is given by

(2) $$E_{s}=\frac{V_{0}}{d_{\mathrm{buf}}}\frac{Z_{\mathrm{buf}}}{Z_{\mathrm{total}}}.$$

$V_{0}$ is the supplied voltage and was set as 44.81 V [42]. For the frequency of 60 kHz, the field strength was found to be $1.33\;{V\;m}^{-1}$, which is 1.5 times smaller as reported earlier [12,13,35,36,37,38]. The deviation can be mainly attributed to incomplete documentation of how the expected $2\;{V\;m}^{-1}$ were determined before [12,13,35,36,37,38], as well as limited knowledge of the prior used geometry. This lack of information led to the suggestion of a novel documentation guideline for ES experiments by our group [46]. In general, the electric field strength in the cell culture medium is a less accurate measure of the stimulation effect than the TMP.

We found that the numerical model of the simplified geometry of the here investigated ES chamber agrees very well with the analytical equivalent circuit expression (1) and (2) (Figures S1 and S2). Moreover, the FEA for the full geometrical model leads to similar results. The impedance deviates less than 1% between the numerical FEA solution and the analytical solution based on equivalent circuits (Figure S3). If one takes only the part of the chamber, where a spatially uniform field exists (i.e., up to a radius of about 10 mm), the electric field strength deviates less than 1% (Figure S4). Thus, the macroscopic electric behaviour of the stimulation device can be characterised solely by equivalent circuits without using FEA. In addition, this result validates the numerical simulation approach demonstrated here.

**Figure 3:**
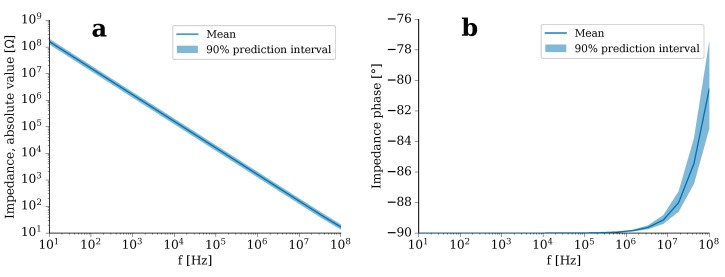
Uncertainty quantification (UQ) result for (**a**) the absolute value and (**b**) the phase of the impedance of the equivalent circuit (Equation (1)). The mean value is shown together with 90% prediction interval for a broad frequency range.

Since each variation of geometrical parameters would require the generation of a new mesh in the FEA, we investigated the effect of geometrical parameters solely for the analytical equivalent circuit model. The UQ analysis shows that the absolute value of the impedance scales linearly with the frequency (see Figure 3a). Its mean is centred in the 90% prediction interval for all frequencies. The phase of the impedance is mostly −90°, which indicates the strong capacitive behaviour of the circuit (see Figure 3b). Only at frequencies greater than 1 MHz does the phase increase slightly. The field also scales linearly with the frequency (not shown).

**Figure 4:**
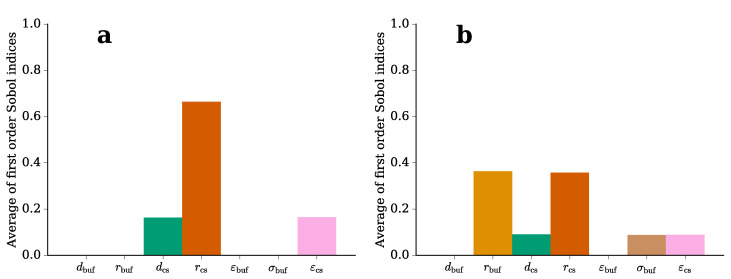
First order Sobol indices for (**a**) the absolute value of the impedance of the equivalent circuit (Equation (1)) and (**b**) for the electric field in the buffer medium (Equation (2)).

The direct influence of each parameter on the model outcome can be expressed by the first order Sobol indices [47]. The Sobol index was computed for each frequency. The results indicate that the Sobol indices do not change with the frequency for the made assumptions (not shown). Hence, we only report the averaged Sobol indices (Figure 4). For the impedance, the geometric parameters of the cover slips (radius and thickness) as well as the permittivity of the cover slips strongly influence the impedance value (Figure 4a). In contrast, the electric field in the buffer medium is also influenced by the radius of the buffer as well as its conductivity (Figure 4b). The uncertainty of the permittivity of the buffer does not play a significant role (Figure 4).

The assumptions for the UQ must be made depending on the prior knowledge about the set-up. We chose uniform distributions for the uncertain parameters reflecting our prior knowledge about the experimental conditions. Nonetheless, our solution can easily be adapted by using one of the different distributions available in Chaospy [48] instead of the uniform distribution chosen by us.

For future experiments with electrically conductive hydrogels and capacitive-coupling stimulation, our findings imply that the dimensions and permittivity of the cover slips should be precisely known as they influence the electric field in the medium and thus the effect of the ES. It is desirable to combine ES and impedance-based metrology [49]. Our results indicate that impedance spectroscopy can be a tool for the experimental validation of the theoretical model. However, it needs very precise knowledge on the stimulation chamber. Its use as a measurement tool is complicated by the impedance of the cover slips, which is much greater than the impedance of the cell buffer. As the hydrogels are expected to have similar properties as the cell culture medium, their properties are hard to be monitored during ES. In addition, the electric field strength can probably not be estimated reliably upon impedance measurement. Nevertheless, capacitive coupling is used in metrology to measure the conductivity of, for example, electrolytes [50,51]. The considerations and analysis tools presented here could be helpful for the design of a novel device which can stimulate and measure the impedance simultaneously. The results indicate that the impedance of the electrode insulation, i.e., the cover slips, has to be substantially decreased by decreasing the insulation’s thickness, increasing its area, or using a high-permittivity material. The latter would also increase the field strength in the medium substantially while requiring a smaller input voltage, which would make the capacitive-coupling approach more energy-efficient [46,52].

### 2.2. A Numerical Model for Cell-Laden Electrically Conductive Hydrogels

We observed the following behaviour in our simulations when we included a hydrogel with variable conductivity (see S11). Regarding different relations of hydrogel conductivity ($\sigma_{\mathrm{hydro}}$) and conductivity of the surrounding cell culture medium ($\sigma_{\mathrm{buf}}$), we conclude three possible outcomes of the ES, which are represented by the electric field inside ($E_{i}$) and outside ($E_{o}$) the scaffold:

1. $\sigma_{\mathrm{hydro}}>\sigma_{\mathrm{buf}}\Rightarrow E_{i}<E_{o}$.
2. $\sigma_{\mathrm{hydro}}<\sigma_{\mathrm{buf}}\Rightarrow E_{i}>E_{o}$.
3. $\sigma_{\mathrm{hydro}}=\sigma_{\mathrm{buf}}\Rightarrow E_{i}=E_{o}$.

The field depends on the hydrogel conductivity for a wide frequency range. Solely at high frequencies, the field is influenced by the hydrogel permittivity and the buffer conductivity. This influence becomes visible by slight changes in the field strength at the edges of the hydrogel, where it is in direct contact with the surrounding medium. We do not expect any influence of this comparably small effect on the cell stimulation. The total impedance of the stimulation chamber is unaffected by a changing hydrogel conductivity or permittivity (Figure S5). In turn, this means that a possible change of the dielectric properties of the hydrogel cannot be monitored in situ.

**Figure 5:**
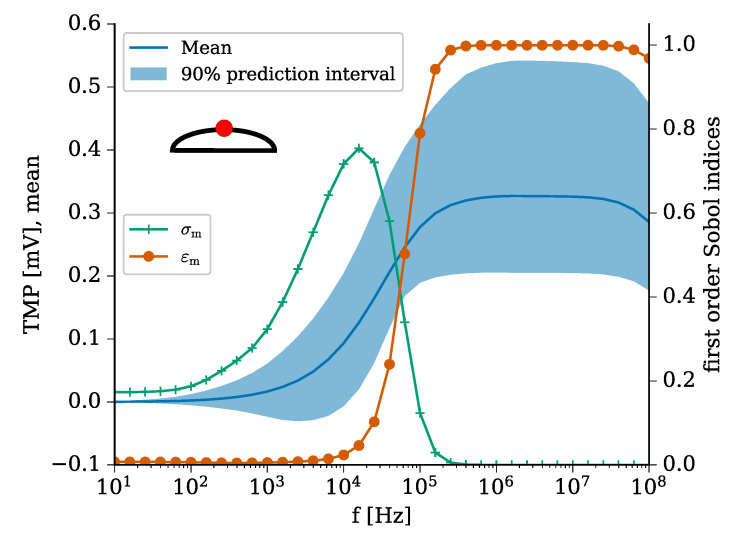
UQ results for the TMP at the cell apex (cell top; red dot) for the benchmark model, where an elliptical cell was assumed to adhere to the bottom of the chamber, representing a 2D cell culture. The mean value is shown together with 90% prediction interval for a broad frequency range (left axis). The first order Sobol indices of the uncertain parameters are shown on the right axis (lines with markers). The parameters, which have Sobol indices less than 0.1 over the entire frequency range, are not shown for the convenience of the reader. These parameters are buffer and cytoplasm conductivity as well as buffer and cytoplasm permittivity. It turns out that the slope of the TMP is mainly defined by the cell membrane conductivity ($\sigma_{m}$) and membrane permittivity ($\varepsilon_{m}$).

Before developing the model of the cell-laden hydrogel, we chose a cell attached to the bottom of the chamber as an established benchmark of an in vitro 2D cell culture [32,53]. As the TMP is greatest at the cell apex (Figure 5, cell top; red dot), we chose this point as the reference configuration for our analysis. Moreover, we did not find any difference between the cell apex and the cell bottom for the benchmark configuration, which relies on [53]. The results of the UQ analysis (Figure 5) reveal that the TMP rises from about 10 kHz on. This frequency mainly depends on the cell membrane conductivity ($\sigma_{m}$). From this frequency on, the magnitude of the TMP depends solely on the membrane permittivity ($\varepsilon_{m}$). Since the other uncertain parameters, including the medium conductivity, did not contribute, we assume that if the cell was covered by a hydrogel, its conductivity would not lead to a changed TMP. This conclusion applies to the case where the conductivity of the hydrogel is in the same range as the tested medium conductivity, i.e., 0.5 to $1.5\;{S\;m}^{-1}$ (Table 1).

**Figure 6:**
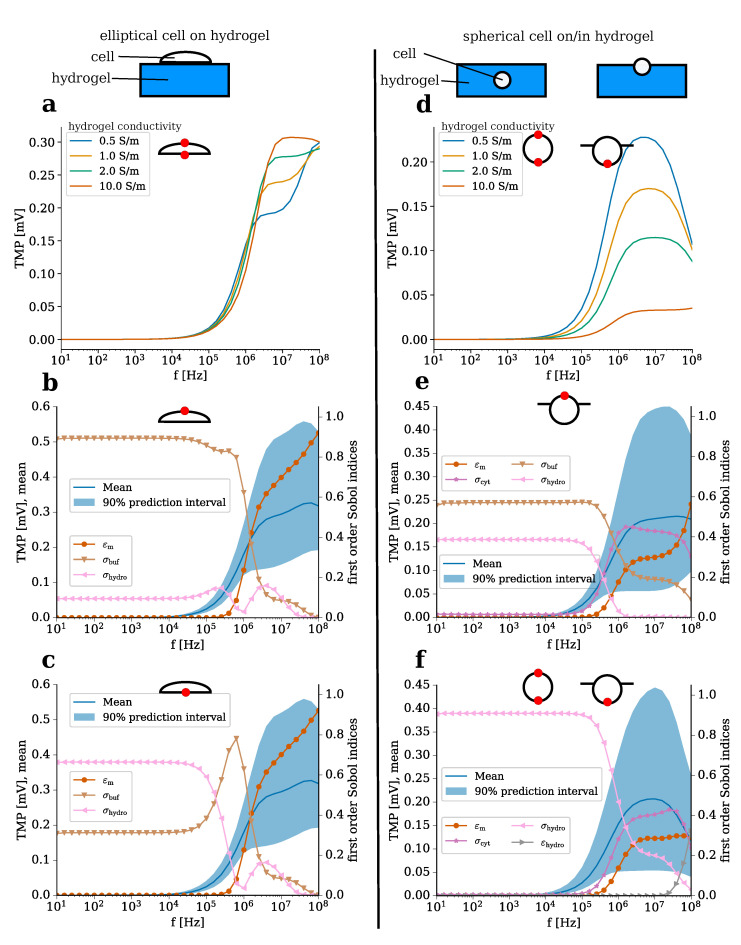
Comparison of an elliptical cell on the top surface of the hydrogel (**a**–**c**) and a spherical cell seeded on/centred in the hydrogel (**d**–**f**). Here, we only report the case where the cell is located at the hydrogel–medium interface since there is a difference between the TMP at the top and bottom of the cell to be expected (Figure S8). The result for a single cell centred in the hydrogel is shown in Figure S9. It almost perfectly resembles (**f**). In each part of the figure, the point on the cell membrane, where the TMP was evaluated (or could have been evaluated yielding similar results in case of the spherical cell), is indicated by a red dot. The TMP for different hydrogel conductivities is compared (**a**,**d**). In (**b**–**f**), the UQ results are shown. The mean and the 90% prediction interval (left axis) are shown together with the first order Sobol indices of the uncertain parameters (right axis). Tested parameters whose Sobol index does not exceed 0.1 over the entire frequency are not shown for the convenience of the reader. Hence, only results for membrane permittivity ($\varepsilon_{m}$), cytoplasm conductivity ($\sigma_{\mathrm{cyt}}$), buffer conductivity ($\sigma_{\mathrm{buf}}$), hydrogel conductivity ($\sigma_{\mathrm{hydro}}$), and permittivity ($\varepsilon_{\mathrm{hydro}}$) are shown.

We investigated the influence of the electrically conductive hydrogel on an exemplary cell. In contrast to the above-mentioned benchmark model, this model corresponds to a 3D cell culture, which mimics the conditions inside the native tissue better. We compared two cell geometries (Figure 6): an elliptical cell on top of the hydrogel (Figure 6a–c), which represents the adhesion step and a spherical cell centred in or placed on top of the hydrogel (Figure 6d–f). The former configuration (cell on top) represents a elongated shape following adhesion on the scaffold [54], while the latter configuration (cell in/on hydrogel) corresponds to a simplication of the cell shape found in articular cartilage [55].

We analysed the individual influence of the hydrogel conductivity. A conductivity of $10\;{S\;m}^{-1}$ was considered as a boundary case, which would correspond to a hydrogel conductivity about ten times greater than the bovine articular cartilage conductivity [33]. In the case of a cell adhering to the hydrogel surface, the hydrogel conductivity does not substantially influence the TMP value over a broad frequency range. The simulation result implies that only in a narrow high-frequency region above 1 MHz does the hydrogel conductivity substantially affect the TMP when subjected to the capacitive electrical stimulation investigated in the present study (Figure 6a).

However, the results indicate that a cell seeded on or placed inside the hydrogel will be stimulated less with increasing hydrogel conductivity (Figure 6b). Only the part of the membrane that is not in direct contact with the hydrogel was not influenced by the hydrogel conductivity (not shown). For both cell geometries, the TMP appears to increase significantly only for frequencies above 100 kHz (Figure 6). As a result, the simulation suggests that cell stimulation is most efficiently influencing cell TMP at frequencies greater than 100 kHz, hence cell stimulation might be optimal in the investigated setup for high frequencies. The main difference (besides their shape) between the two cell configurations considered here is their exposure to the hydrogel. While the adherent, elliptical cell only touches the hydrogel surface, the spherical cell is embedded in the hydrogel (Figure 6). Thus, the smaller influence of the hydrogel conductivity on the TMP of the elliptical cell appears to be logical.

By means of UQ, the influence of the other parameters and their uncertainties on the possible TMP values was studied (Figure 6b–f). For the elliptical cell, the TMP depends mostly on the membrane permittivity in these frequency regions (Figure 6b,c). In general, both sides of the cell experience a similar change in the TMP. The conductivities of the adjacent buffer medium (cell apex) or hydrogel (cell bottom) do not contribute much to the rise of the TMP.

The membrane permittivity also plays a role for the spherical cell (Figure 6e,f). In addition, the conductivities of the environment, i.e., of the buffer medium (cell apex) as well as of the hydrogel (cell bottom), influence the TMP (Figure 6e,f). As could be expected, the membrane region exposed to the cell culture medium is influenced more by the conductivity of the culture medium than by the hydrogel conductivity. In contrast, the membrane region that is exposed to the hydrogel is not influenced by the cell culture medium. In the case of a cell centred in the hydrogel, only the conductivity of the hydrogel has an impact on the TMP (Figure S9). In the region above 100 kHz, where the TMP rises, the influence of the environment decreases and the influence of the cell dielectric properties—for example, membrane permittivity and cytoplasm conductivity—increases (Figure 6e,f). The influence of the cytoplasm conductivity cannot be observed for the elliptical cell (Figure 6b,c). Furthermore, we found that the TMP of a cell close to the hydrogel–medium interface resembles the TMP of a cell centred in the hydrogel (compare Figure S9 and Figure 6f). The 90% prediction interval can become very broad for both cell shapes and extends up to 40 mV. In the regions where the prediction interval is broad, the variance of the TMP is mainly caused by the cellular dielectric properties.

In comparison to the benchmark of a single adherent cell (Figure 5), it becomes evident that the TMP of a cell on/in a hydrogel can reach a similar magnitude. However, this magnitude is only reached at higher frequencies greater than 100 kHz. For an adherent cell, the TMP is mostly influenced by the dielectric properties. Hence, it cannot directly be influenced by varying the conductivity of the cell culture medium. Using electrically conductive hydrogels, it becomes feasible to optimise the effect of the ES for cells seeded on or embedded in the hydrogel. Altering the electrical conductivity of the hydrogel to values lower than the surrounding medium could be a tool to increase the TMP while higher conductivity values would cause a decrease of the TMP.

In a recent study, a homogeneous capacitively coupled electric field of $1\;{V\;m}^{-1}$ at 60 kHz has been shown to increase the synthesis of chondrogenic markers of mesenchymal stem cells embedded in injectable hydrogels [11]. Another study investigated the effect of smaller capacitively coupled fields in the range of $\mu {V\;m}^{-1}$ at 60 kHz on chondrocytes seeded on collagen type I hydrogels. The underlying mechanism of the ES by capacitively coupled fields at 60 kHz has been studied by Xu et al. [37]. They observed an up-regulation of chondrogenic ECM molecules (collagen type II, aggrecan) unless voltage-gated channels were inhibited. These channels cause an influx of extracellular Ca^2+^ when triggered, which has been linked to the effect of ES [37]. However, Xu et al. studied chondrocytes seeded on cover slips, which corresponds to a 2D cell culture, i.e., the benchmark problem of an elliptical cell adhering to the bottom of the ES chamber (Figure 5). Voltage-gated channels are activated by changes in the TMP. In general, a change in the TMP ranging between $10^{0}$ mV [43] and 10^2^ mV [40] has been estimated to be sufficient for a significant effect. While we found an increased TMP at 60 kHz for the benchmark model, but not for cells in contact with hydrogels, an effect on cells inside a hydrogel due to a change in the TMP seems unlikely. Frequencies, for which we found the TMP to reach biologically relevant values in the mV range, have yet not been considered in CTE. They could be tried in the future when conducting experiments with electrically conductive hydrogels. A frequency of about 5 MHz could be optimal to verify the hypothesis that an induced change in the TMP causes the biological effect referring to the reported increased ECM synthesis [12,13,14], improved re-differentation of de-differentiated cells [9,10], and the enhanced chondrogenic differentiation [11].

However, care must be taken of the cell line and its dielectric properties. The change in the TMP due to ES is significantly influenced particularly by the dielectric properties of the cell membrane. In future research, differences between different cell lines with respect to their dielectric properties could be investigated, for example, using dielectric spectroscopy [56]. The conductivity of the hydrogels can be tuned to test the hypothesis that indeed a lower hydrogel conductivity leads to a greater change in the TMP and in turn to an increased effect of the ES.

Our results indicate that there is no crucial difference of the stimulation with respect to the cell location inside the hydrogel and the cell morphology. Nevertheless, for future research, we aim at developing more realistic models with many cells as recently presented for a different use case [57]. However, our study contributes to the design of future UQ studies for such complex and numerically expensive models. In the present work, we could determine the sensitivity of the model with respect to the different modelling parameters and do not expect a general change of the underlying physics. Hence, different parameters that have not revealed a great influence on the model outcome do not need to be considered in the future. This decreases the complexity and numerical cost of future UQ studies substantially.

### 2.3. Theoretical Considerations Regarding Cellular Organisation

Cellular organisation inside hydrogels can be controlled by an external electric field [58]. For alternating current fields, the force acting on the cells depends on the gradient of the electric field strength and the real part of the so-called Clausius–Mossotti (CM) factor [59]. This phenomenon is referred to as dielectrophoresis. In homogeneous fields as they occur in the set-up discussed in the previous section, no cellular organisation is to be expected. A net force guiding the cells occurs only in non-uniform fields. Devices generating such fields are currently under development for CTE using capacitive coupling [60]. Nevertheless, cells can deform due to the electric field [61,62]. Recently, mesenchymal stem cells stimulated by EFs of $1\;{V\;m}^{-1}$ at 60 kHz have been found to become rounder upon ES [11].

**Figure 7:**
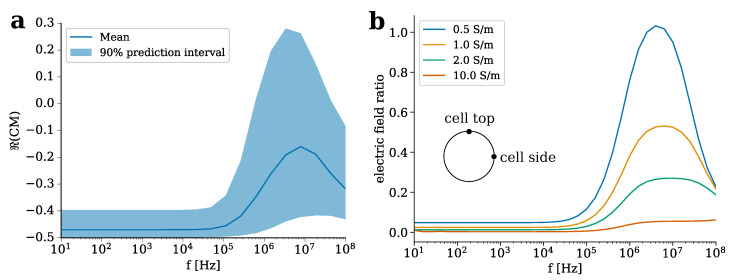
(**a**) Mean value and 90% prediction interval for the real part of the CM factor, ℜ(CM). (**b**) Ratio between the field at the top and at the side of a cell, which is centred in the hydrogel, for different conductivities. This means that the field is always greater at the side of the cell when the ratio is less than one.

We investigated the effect of the hydrogel properties on the real part of the CM factor (Figure 7). This quantity can be understood as a relative force, the sign of which indicates whether a cell would experience a force in direction of lower (negative sign) or higher (positive sign) field strengths. The cell deformation would also follow this force [62,63,64]. Considering the theoretically possible values for eukaryotic cells [65], which includes chondrocytes, and the desired values for hydrogels (see Table 1), we find that the mean value of the real part of the CM factor remains negative for most of the frequencies (see Figure 7a). Only between 1 MHz and 100 MHz, the probability that it assumes a positive value is largely increased as its value may become greater than zero. In this frequency window, the permittivity of the membrane and the conductivities of the hydrogel and the cytoplasm need to be known precisely to determine the sign of the CM factor reliably (see also Figure S10).

If the electric field of the external medium is homogeneous, the field distribution inside the hydrogel is also mostly homogeneous. Then, in a uniform field as considered here, solely the poles of the field around the cell membrane matter [62]. Thus, we introduced the ratio of the field at the side and the field at the top or bottom of the cell as a measure for the possible deformation of the cell. We studied the influence of the hydrogel conductivity on the field ratio for a spherical cell centred in an electrically conductive hydrogel (Figure 7b). The ratio is close to zero up to 100 kHz, indicating that the field at the side of the cell is much greater than at the top of the cell. In this frequency range, the ratio decreases with increasing conductivity. Above 100 kHz, the ratio also depends on the hydrogel conductivity. For highly conductive hydrogels, the ratio does not increase considerably. The lower the hydrogel conductivity becomes, the more a peak at about 10 MHz arises. For the lowest conductivity tested, the ratio at this peak becomes one, i.e., the field strengths at cell top and cell side become equal.

The negative real part of the CM factor indicates that it is very likely for eukaryotic cells immersed in electrically conductive hydrogels to be attracted by regions of lower field strengths. As we highlighted in the previous section, hydrogels, which have a conductivity less than the surrounding cell culture medium, will be regions of higher field strengths. Hence, cells seeded on the top side of the hydrogel might experience a repelling force that prevents them from growing into the scaffold. Cells inside the scaffold might also be guided to certain regions of the hydrogel if the externally applied field is non-uniform.

The results for the CM factor and the field ratio (Figure 7) lead to the conclusion that in the case of uniform fields, the cells might experience a force that stretches them perpendicular to the external field vector. Note that due to parallel-plate geometry of the set-up studied by us, the field vector points from cell bottom to cell top. A hydrogel conductivity similar to the cell culture medium or even greater could increase the mechanical effect since it contributes to a smaller field ratio. The mechanical deformation perpendicular to the field could be a reason for the round cell shape since it counteracts adhesion to the hydrogel fibres that are aligned along the field vector. Furthermore, it could lead to the activation of stretch-activated channels, which has been observed in ES experiments with physiological inhibitors [40,66]. Future experimental research is needed to analyse if the force due to the external field is sufficiently large to substantially affect the cells, i.e., if cell migration or deformation occur. The main differences of the stimulation chamber considered here and set-ups to rapidly organize cells is the exposure time and field strength. While the cellular organisation [58] or deformation [63,64] takes place in a few minutes and uses field strengths in the range of $k{V\;m}^{-1}$ or even higher, the stimulation of cell cultures usually happens for considerably longer period of times and is applied repeatedly at field strengths of ${V\;m}^{-1}$ [11,12,37]. Thus, the forces are expected to be at least one order of magnitude smaller but act continuously. Likewise, more sophisticated models of the electromechanobiology of cells and tissues need to be found to eventually predict the deformation or displacement of cells in their native environment [67]. Such models could then explain, for example, why and how a certain channel size in printed scaffolds influences the cell shape [68]. Eventually, an optimal channel width should be determined to promote chondrogenesis, which goes along with spherical cell shape. Due to the rather complex microstructure of the hydrogels and the biological interaction of the cells with the hydrogel fibres, this task currently seems to not be accomplishable with the existing models.

Furthermore, models are needed for the dynamic behaviour of the membrane since due to its polarisation, constituents of the membrane can rearrange under the influence of the external field [69,70]. At the interface of these open fields, optical methods such as confocal laser scanning microscopy of cell-laden hydrogels will play an important role. They allow one to determine the cells’ locations and morphology, which can simultaneously be used to generate geometrical models for FEA [71] and to monitor the effect of the ES. Our theoretical approach shows that hydrogel conductivity can alter the forces superimposed on the cells by ES, leading to higher forces on the cells when embedded in hydrogels with higher conductivity than the surrounding medium. Gels with higher conductivity could facilitate cellular ingrowth and promote round cell shapes due to this force. However, the actual net force cannot be reliably estimated and might require considerably higher field strengths in the order of $k{V\;m}^{-1}$ to take effect.

Another possible effect of the electric field could be heating. The frequency of 60 kHz falls into the radiofrequency range. Frequencies in this range (however usually about 500 kHz) are used for thermal tissue ablation [72]. The thermal effect of the electric field in this frequency range is mainly influenced by the conductivity [73]. Considering the conductivity of the cell culture medium, its volume and the reported field strength, the applied power is in the range of a few μW. This is far below the usually applied power of considerably more than 1W [72,73]. Thus, no significant thermal effect of the electric field is to be expected.

The main implications of our theoretical work for future experimental studies concern the stimulation device and the conductivity of the hydrogels for CTE. In general, devices for capacitive-coupling stimulation should have a decreased impedance compared to the device studied here. This would enable energy-efficient stimulation and potentially permit in situ monitoring. The choice of the stimulation frequency depends on the desired effect on the cells. If the TMP of the cells shall be influenced by ES, frequencies above 1 MHz could be explored. For a mechanical effect on the cells, lower frequencies in the range, which is currently used, could be chosen. The conductivity of the hydrogels can be chosen accordingly. Our results suggest that a conductivity less than the cell culture medium conductivity could beneficially contribute to an increased change in the TMP. In contrast, the mechanical effect could be promoted by hydrogels with a conductivity greater than the cell culture medium conductivity.

One limitation of our study is that the chemical cell–hydrogel interaction has not been taken into account. A goal of future research is to predict biocompatibility of hydrogel materials, which always has to be ensured in CTE, by theoretical means. Theoretically, the conductivity of the scaffold can be increased by increasing the concentration of conductive fillers such as polypyrrole, (reduced) GO (rGO) or carbon nanotubes [16]. In practice, dose-dependent cytotoxic effects have been observed when using carbon nanotubes [74] or other nanoparticulate conductive polymer fillers [75,76]. For oligopyrrole micronetworks inside hydrogels [25] or rGO containing hydrogels [21,24], cytocompatibility and in-vivo compatibility has been shown in canine animal models [21,24] for CTE. Among the studies with conductive hydrogels for application in CTE, hydrogels having partly or fully reduced GO allowed for the highest conductivity values greater than $1\;{S\;m}^{-1}$ [24] and even up to $10\;{S\;m}^{-1}$ [21] while being biocompatible. Polypyrrole hydrogels for CTE were fabricated with a low conductivity less than $0.5\;{S\;m}^{-1}$ [25]. Hence, they have been less conductive than native bovine articular cartilage, which has a conductivity of about $1\;{S\;m}^{-1}$ [33]. The studies we referred to here have focused on a limited amount of synthetic and natural-based polymers [21,24] and chitosan-based [25] scaffolds. However, among the plethora of scaffold materials tested for general CTE [18], there might be more suitable candidates for electrically conductive hydrogels and ES-assisted CTE. This has to be addressed in future research, while our study provides a theoretical framework which might help to guide the development and design of future electroactive hydrogel scaffolds for ES-assisted CTE.

Furthermore, increasing the conductivity of the hydrogel might lead to a changed osmolarity that is caused by an increased fixed charge density in the hydrogel [77]. Chondrocytes react to changes in osmolarity [78], which might mask the effect of the ES, and may give in combination with ES an exciting field for future research of ES-assisted CTE using electroactive hydrogels.

## 3. Materials and Methods

### 3.1. Geometric Modelling and Equivalent Circuits

As the stimulation device by Brighton et al. [35,42] has heavily influenced the field of ES in TE, we chose it as a reference for our geometry model. Based on the original publications, we tried to recreate the original geometry of the device. The assumptions made to generate the geometry are in detail given in Appendix A. In total, this approach yields the geometrical model shown in Figure 2. For the sake of numerical efficiency, we chose an axisymmetric representation of the geometry. Furthermore, we did not model the electrodes explicitly. Instead, we assigned a fixed value of the potential to the sides of the cover slips that are not in contact with the cell culture medium. Thereby, we achieve capacitive coupling, since there is no direct current flow from the electrodes into the medium. When considering a hydrogel, we added a domain with 1 mm radius and 1 mm height at the symmetry axis on the bottom cover slip (see also Figure S8). The geometry Figure 2 can be reduced to a parallel-plate-capacitor geometry, where the influence of the area with the plastic lid and dish with the contained medium is neglected. In this case, the (almost) perfectly insulating glass cover slips can be understood as capacitors and the buffer medium as a lossy dielectric. A lossy dielectric can be described by the complex permittivity $\varepsilon^{*}=\varepsilon_{r}\varepsilon_{0}-i\sigma /\omega$, which comprises relative permittivity $\varepsilon_{r}$, vacuum permittivity $\varepsilon_{0}$, conductivity σ, and angular frequency ω. This complex permittivity can be understood as an RC circuit [45]. Under the assumption of an idealized parallel-plate capacitor, the impedance for each part becomes

(3) $$Z=\frac{1}{i\omega C}=\frac{d}{i\omega \varepsilon^{*}A},$$

where *d* is the thickness of the layer (cover slips or medium) and *A* is the cross-sectional area. We assume for the cover slips a thickness of $d_{\mathrm{cs}}=0.15$ mm and a radius $r_{\mathrm{cs}}=16.5$ mm. The medium has a thickness $d_{\mathrm{buf}}=1.415$ mm. Furthermore, we assume it to have a radius $r_{\mathrm{buf}}=r_{\mathrm{cs}}$. The dielectric properties of cover slips and medium, respectively, are denoted by subscripts cs and buf. Eventually, the total impedance is the sum of the individual impedances. The field inside the cell culture medium can also be estimated. The circuit acts as a voltage divider. Hence, the voltage in the medium is

(4) $$V_{\mathrm{buf}}=V_{0}\frac{Z_{\mathrm{buf}}}{Z_{\mathrm{total}}}.$$

Then, the field is (assuming a parallel plate capacitor)

(5) $$E_{s}=\frac{V_{\mathrm{buf}}}{d_{\mathrm{buf}}}.$$

Here, $V_{0}$ is the amplitude of the initially applied voltage. We chose frequencies between 10 Hz and 100 MHz since they could be covered by commercially available impedance analysers [79]. Moreover, in this frequency range wave effects, which we neglected in our analysis, are not to be expected [80].

### 3.2. Finite Element Analysis and Uncertainty Quantification

As the field might become non-uniform when a hydrogel is considered, we opted for FEA to compute the electric field and the cell’s TMP. We chose a modelling approach that is in detail described elsewhere [81]. Briefly, we solved the electro-quasistatic representation of Maxwell’s equations [80] by COMSOL Multiphysics^®^, V5.3a employing second-order Lagrange elements and a direct solver.

We studied different configurations of the cells. The benchmark configuration is an elliptical cell attached to the top of the bottom cover slip (Figure S6). The same cell is placed on top of the hydrogel (Figure S7). In addition, we studied two spherical cell configurations (Figure S8). Firstly, we placed the cell on the top of the hydrogel such that parts of it are surrounded by the medium and parts of it by the hydrogel. Secondly, we assumed a spherical cell at the center of the scaffold. Since the field around biological cells in a homogeneous medium has two poles (Figure S8a), we identified the position of these poles by computing the field strengths at top, bottom, and side of the cells (Figures S8a, S12 and S13). The parameters we assume for the numerical computation of the electric potential are listed in Table 2. They are primarily based on [32], which is an established benchmark for the estimation of the effect of the ES.

Since we found previously a large dependency of the model outcome on the initial parameter assumptions [81], we again applied UQ techniques. They can be distinguished into two groups: Monte Carlo (MC) [82] and Polynomial Chaos (PC) [83] techniques. While in MC techniques the parameter space spanned by the probability distributions of the individual parameters is sampled, PC techniques rely on a surrogate model. This model is built based on the assumed probability distributions and contains all information about the physical model and its uncertainty. Both methods are featured in the open-source Python code *Uncertainpy* [84]. We used a modified version of the source code (https://github.com/j-zimmermann/uncertainpy/tree/1.2.0.1) for our UQ computations. For the FEA, we used PC techniques for the UQ analysis. The configuration was the same as in previous research [81], where we established the methodology to propagate the cell dielectric parameters’ uncertainty through the computational model. For the equivalent circuit models and other models based on analytical equations, which are not numerically expensive to evaluate, we relied on MC techniques. They require more model evaluations, i.e., function calls but do not rely on a surrogate model, and thus make overall fewer assumptions about the system [85]. For example, for six uncertain parameters, we needed 422 function calls with PC techniques but 40,000 with MC techniques.

The choice of the probability distributions reflect the a priori knowledge of the modeller. For the geometrical parameters, we made the assumption that they vary uniformly by 10% around the mean value. In contrast to the cell culture medium, which is considered in models without hydrogels, the relative permittivity of the hydrogel is not known. For cartilage, it can easily increase up to 10^3^ [86]. Thus, we considered a broad span from the cell culture medium case to the cartilage case in our UQ analysis of the hydrogel model (see Table 1).

To estimate the relative force acting on the cells inside a hydrogel, we chose the formulation of the CM factor, which is valid for spherical cells

(6) $$\mathrm{CM}=\frac{\varepsilon_{2}^{*}-\varepsilon_{1}^{*}}{\varepsilon_{2}^{*}+2\varepsilon_{1}^{*}},$$

where $\varepsilon_{1}^{*}$ is the complex permittivity of the surrounding medium, i.e., the hydrogel, and $\varepsilon_{2}^{*}$ is the complex permittivity of the cell. For spherical eukaryotic cells, there exists an analytical formula to compute $\varepsilon_{2}^{*}$ with known experiential limits of the model parameters [65]. These limits (Table 3 and Table 4) serve as the input of our UQ study. For the hydrogel, we assumed the aforementioned lossy dielectric.

## 4. Conclusions

Even though this study is purely theoretical, we could highlight different aspects that are highly relevant for the design of ES experiments with electrically conductive hydrogels for CTE. While the hydrogel conductivity was shown to influence the outcome of the stimulation quite crucially in different aspects, the hydrogel permittivity did not have such an impact.

Based on this study, two future directions for enhancing the understanding of capacitive-coupling stimulation of chondrocytes seeded on hydrogels can be pursued:

1. Development of chemically and electrically stable low-conductivity hydrogels (<$1.5\;{S\;m}^{-1}$) to increase the electrical field strength acting on the chondrocytes and thus increased change in their TMP. Here, low-impedance insulation of the electrodes from the cell culture medium becomes highly relevant to ensure an electrically efficient solution. Possible hydrogel materials could be, for example, solely ionically conductive hydrogels [7] or a suitable hydrogel functionalised with, for example, polypyrrole [25] or reduced graphene oxide [24].
2. Usage of high-conductivity hydrogels (>$1.5\;{S\;m}^{-1}$) together with non-uniform, strong electric fields much greater than $1\;{V\;m}^{-1}$ to exploit the attraction of chondrocytes by low-field regions. This could be used to influence cellular ingrowth inside hydrogels and increase efficiency of initial cell seeding. Such high conductivities might only be reached by the use of, for example, highly conductive carbon nanotubes [20], reduced graphene oxide [21,24], or well-percolating doped conductive polymer networks made from, among others, polypyrrole or polyaniline [15,19].

However, it cannot be excluded that another effect, which was not covered by this study, occurs during ES and affects the cells. Since our approach is easily reusable for other numerical models of ES experiments, we expect that our study will contribute to future research in this field. In particular, the inclusion of parameter uncertainties or possible parameter ranges into the modelling workflow allows experimenters to find optimal parameters and efficient experiment designs.

## Figures and Tables

**Table 1:** Assumptions for the dielectric properties of electrically conductive hydrogels with an exemplary single cell model [32]. The uniform distribution is denoted by U.

Domain (Subscript)	Conductivity σ[S m${}^{-1}$]	Permittivity ε
Hydrogel (*hydro*)	U(0.1,2.0)	$U(60,1\cdot 10^{3})$
Buffer medium (*buf*)	U(0.5,1.5)	U(60,80) (benchmark) or 80
Membrane (*m*)	$U(0,5\cdot 10^{-5})$	U(5,15)
Cytoplasm (*cyt*)	U(0.1,1.0)	60

**Table 2:** Parameters for the numerical model based on [32].

Domain	Subscript	Electrical Conductivity [S m${}^{-1}$]	Rel. Permittivity
Insulator/Lid	*ins*	0	2.6
Cover slip	*cs*	0	4
Culture medium	*buf*	1.5	80
Cytoplasm	*cyt*	1.5	60
Cell membrane	*m*	0	11.3

**Table 3:** Assumptions for the dielectric properties of eukaryotic cells as reported in [65]. The uniform distribution is denoted by U.

Domain (Subscript)	Conductivity σ[S m${}^{-1}$]	Permittivity ε
Membrane (*m*)	$U(8\cdot 10^{-8},5.6\cdot 10^{-5})$	U(1.4,16.8)
Cytoplasm (*cyt*)	U(0.033,1.1)	U(60,77)
Nuclear envelope (*ne*)	$U(8.3\cdot 10^{-5},7\cdot 10^{-3})$	U(6.8,100)
Nucleoplasm (*np*)	U(0.25,2.2)	U(32,300)

**Table 4:** Assumptions for the geometric properties of eukaryotic cells as reported in [65]. Note that instead of the explicit nucleus radius $R_{n}$, a scale parameter was introduced such that $R_{n}=\mathrm{scale}\;\cdot \;R_{c}$. This ensures that the nucleus radius is always less than the cell radius. The uniform distribution is denoted by U.

Parameter	Symbol	Probability Distribution
Cell radius	$R_{c}$	U(3.5,10.5)μm
Membrane thickness	$d_{m}$	U(3.5,10.5)nm
scale	scale	U(0.28,0.84)
Nuclear envelope thickness	$d_{n}$	U(20,60)nm

## Supplementary Materials

The following are available online, Figure S1: Comparison of electric field strengths of numerical and analytical model. Figure S2: Comparison of impedance magnitudes of numerical and analytical model. Figure S3: Comparison of impedance magnitudes of full-geometry numerical and analytical model. Figure S4: Comparison of electric field strengths of full-geometry numerical and analytical model. Figure S5: UQ result for impedance. Figure S6: Geometrical model of cell adhering to cover slip. Figure S7: Geometrical model of hydrogel in ES stimulation device. Figure S8: Electric field around cells for two different configurations. Figure S9: UQ result for the TMP of a spherical cell centred in the hydrogel. Figure S10: UQ result for real part of CM factor. Video S11: Animation of electric field strength for different hydrogel conductivities. Video S12: Electric field strengths around cell centred in hydrogel at different frequencies. Video S13: Electric field strengths around cell seeded on hydrogel at different frequencies.

## Abbreviations

The following abbreviations are used in this manuscript:

UQ	Uncertainty Quantification
FEA	Finite Element Analysis
ECM	Extracellular Matrix
TMP	Transmembrane Potential
CM	Clausius-Mossotti
MC	Monte Carlo
PC	Polynomial Chaos
TE	Tissue Engineering
CTE	Cartilage Tissue Engineering
ES	Electrical Stimulation
GO	Graphene Oxide

## Appendix A

Based on the original report [87], where the Cooper dish was introduced, we aimed to create a realistic model of the ES system. We assumed a dish with a radius of 30 mm and a recessed area with a radius of 20 mm. The distance from the top of the lid to the inner bottom side of the dish is 2.6 mm. It is mentioned that the volume filling the space between the inner side of the lid and the inner bottom side is 4 mL. This volume must be a cylinder with radius 30 mm and height being equal to the spacing between bottom and inner side of the lid. Hence, the spacing between the inner top and bottom side is

(A1) $$d=\frac{4\mathrm{ml}}{\pi {\left(30\mathrm{mm}\right)}^{2}}\approx 1.415\mathrm{mm}.$$

Furthermore, the thickness of the plastic lid is then known to be

(A2) $$d_{\mathrm{lid}}=2.6\mathrm{mm}-1.415\mathrm{mm}=1.185\mathrm{mm}.$$

Since the cover slips are attached to the well from the outside, the spacing between the cover slips is

(A3) $$d_{s}=1.415\mathrm{mm}+2\;\cdot \;1.185\mathrm{mm}=3.785\mathrm{mm}.$$

The height of the dish has not been mentioned. However, under the assumption that the recessed area is linearly connected to the outer part of the dish and the information that the volume inside the dish is about 7 mL, the volume of a conical frustum with unknown height *h* and known radii r=30 mm on top and R=20 mm on bottom subtracted from the volume of a cylinder of radius 30 mm and unknown height *h* must be equal to the difference of the volumes, i.e., V=3 mL. This yields

(A4) $$h=\frac{V}{\pi \left(r^{2}-\frac{1}{3}\left(r^{2}+R\;\cdot \;r+R^{2}\right)\right)}\approx 3.581\mathrm{mm}.$$

Eventually, the total height of the dish must be

(A5) $$h_{\mathrm{total}}=1.415\mathrm{mm}+3.581\mathrm{mm}=4.996\mathrm{mm}.$$

The dish is filled by 7 mL cell culture medium, i.e., up to the total height of about 5 mm [42]. In contradiction to that, it has been reported earlier that only the space between the two cover slips was filled [35]. As the electric field is of interest between the two plates and the different fill level would only affect regions far away from there, we assume the dish to be entirely filled.
