# Supplementary material for: Numerical Simulations as Means for Tailoring Electrically Conductive Hydrogels towards Cartilage Tissue Engineering by Electrical Stimulation
Source: Molecules. 2020 Oct 16;25(20):4750. doi: 10.3390/molecules25204750 (PMC7587583; doi:10.3390/molecules25204750)
Supplement: Supplementary file 1 [file molecules-25-04750-s001.zip › SI/Zimmermann_SI.pdf]

Supplementary Materials: Numerical simulations as means for tailoring electrically conductive hydrogels towards cartilage tissue engineering by electrical stimulation

Julius Zimmermann [1,*], Thomas Distler [2], Aldo R. Boccaccini [2] and Ursula van Rienen[1,3]

1. Comparison of the analytical and the numerical model

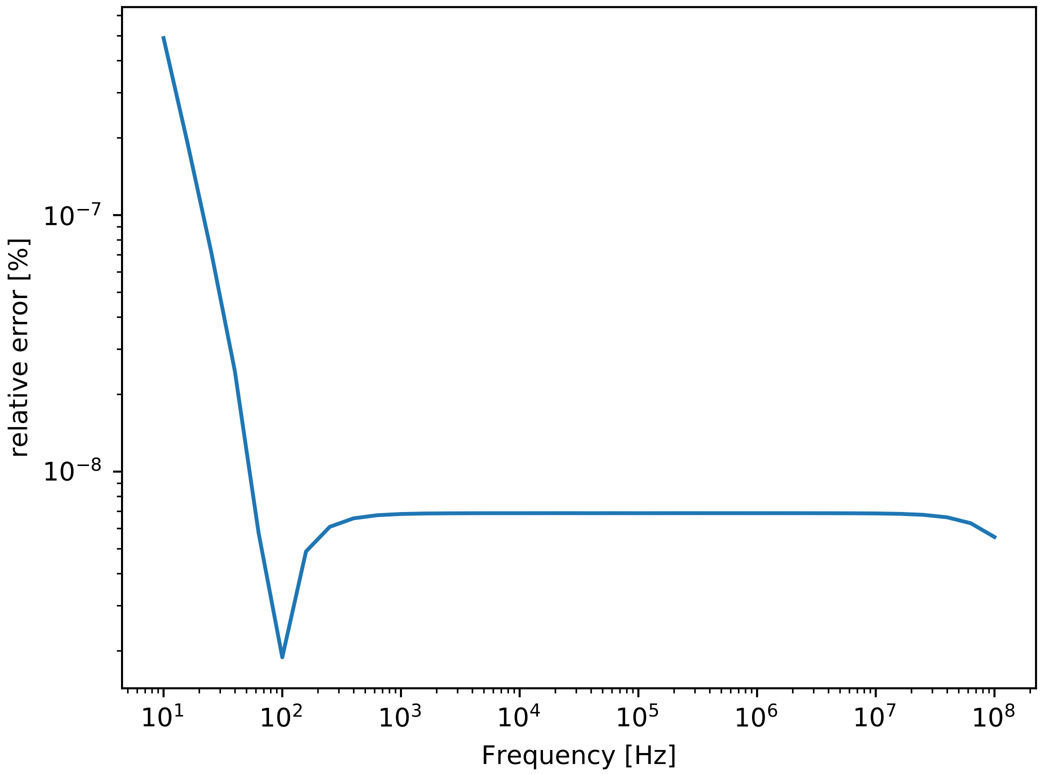

Figure S1. Relative error of the electric field strength inside the cell culture medium between the numerical solution for a simplified geometry compared to the analytical solution based on equivalent circuit analysis.

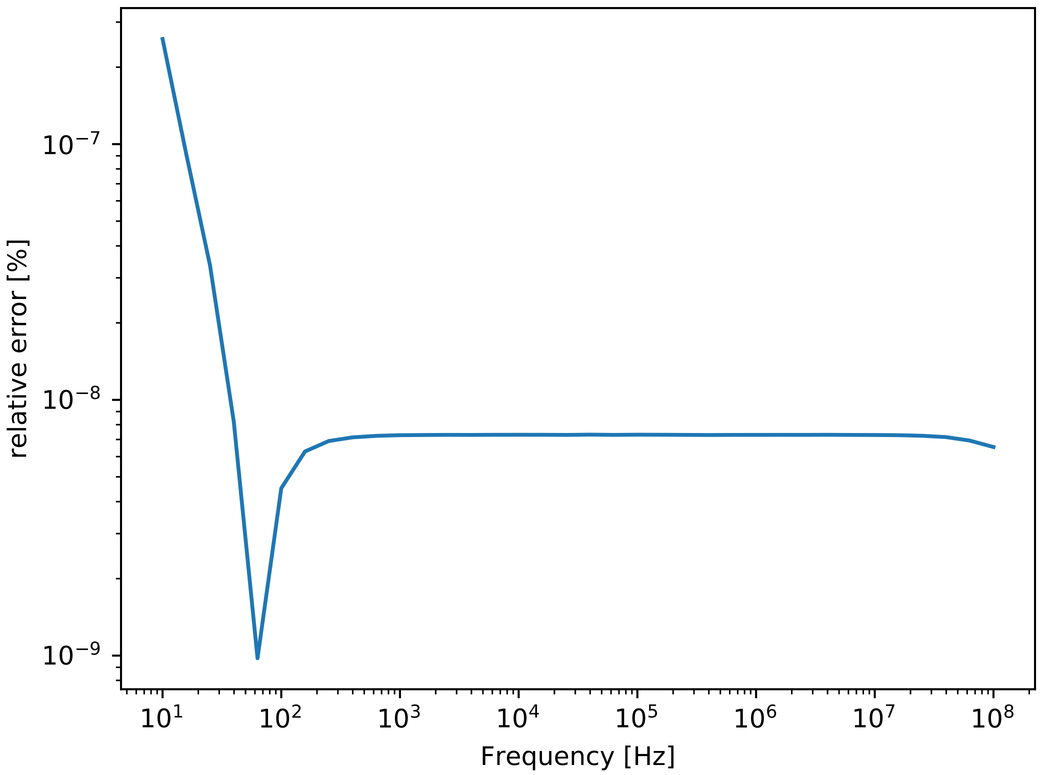

Figure S2. Relative error of the absolute value of the impedance between the numerical solution for a simplified geometry compared to the analytical solution based on equivalent circuit analysis.

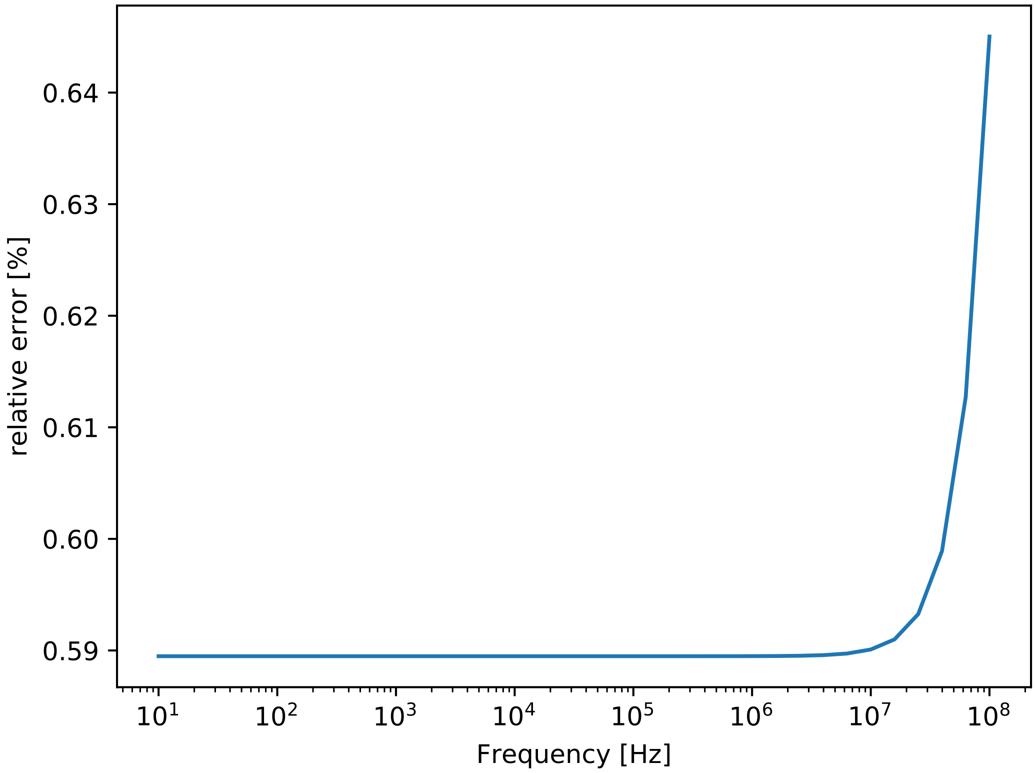

Figure S3. Relative error of the absolute value of the impedance between the numerical solution for the full geometry compared to the analytical solution based on equivalent circuit analysis.

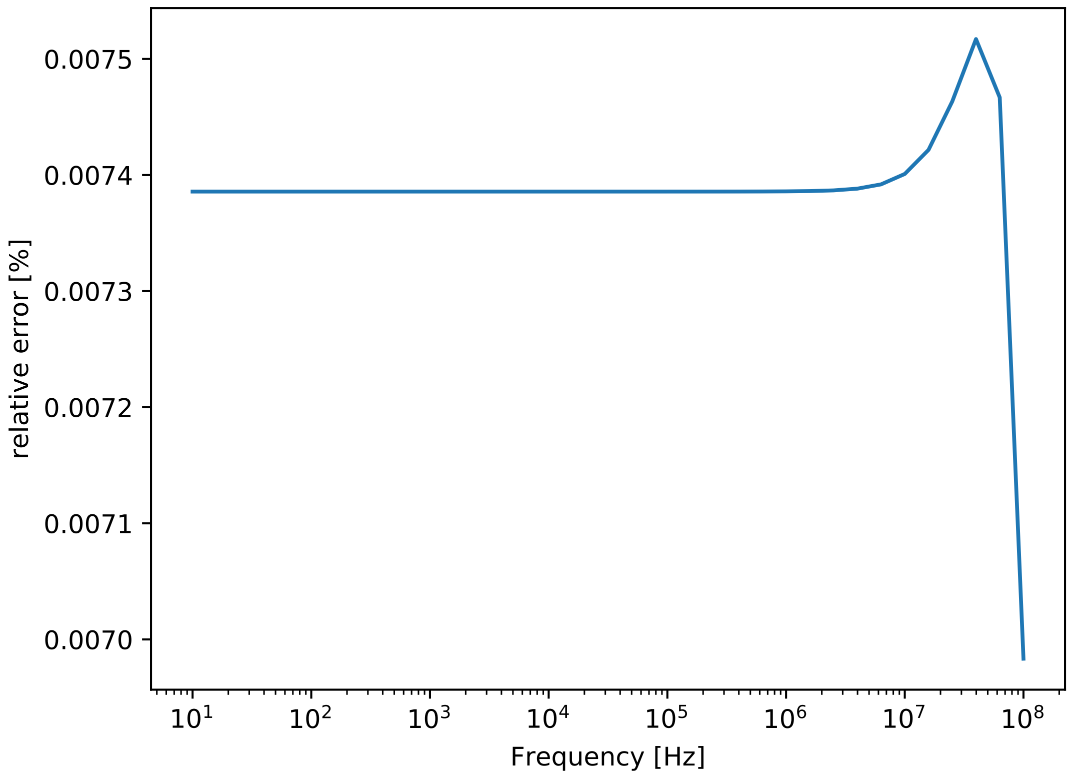

Figure S4. Relative error of the electric field strength inside the cell culture medium between the numerical solution for the full geometry compared to the analytical solution based on equivalent circuit analysis.

2 **2. Uncertainty Quantification result for the impedance**

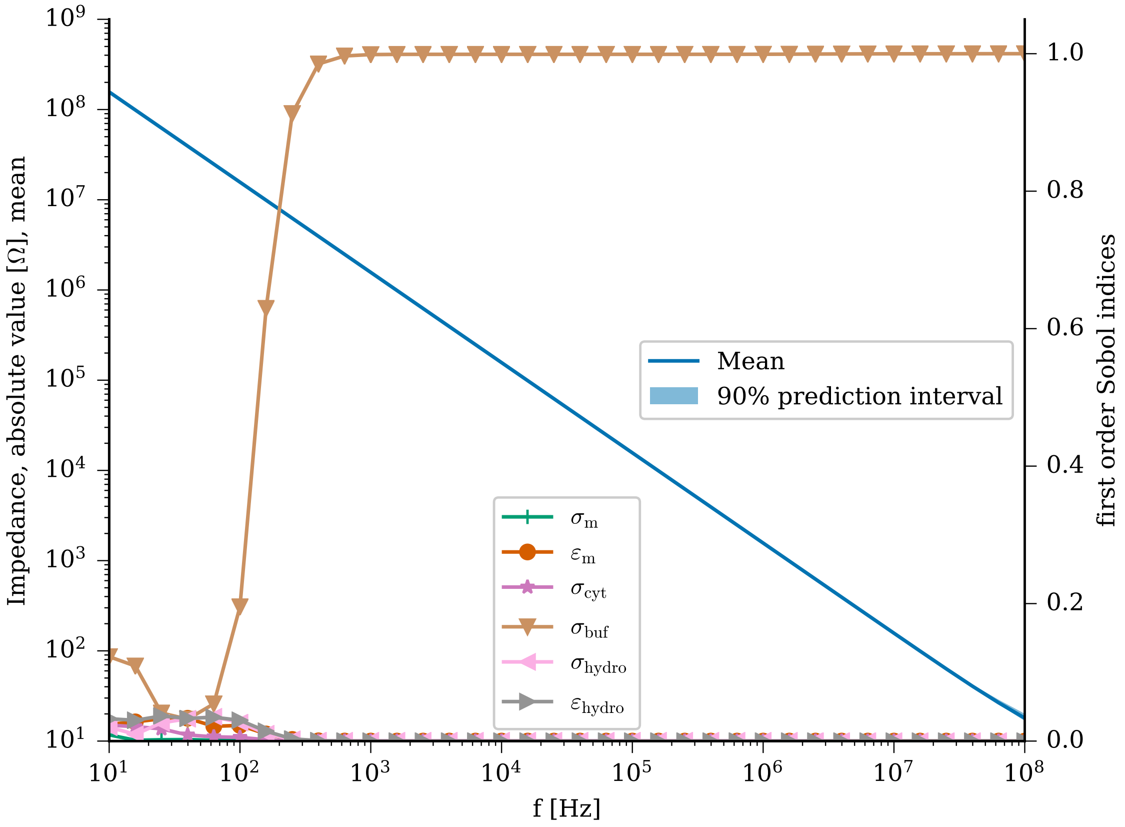

Figure S5. The mean value of the impedance is shown together with 90% prediction interval for a broad frequency range (left axis). The first order Sobol indices of the uncertain parameters (membrane conductivity σ_{m} and permittivity ε_{m}; cytoplasm conductivity σ_{cyt}; buffer conductivity σ_{buf}; hydrogel conductivity σ_{hydro} and permittivity ε_{m}) are shown on the right axis.

3 **3. Cell models**

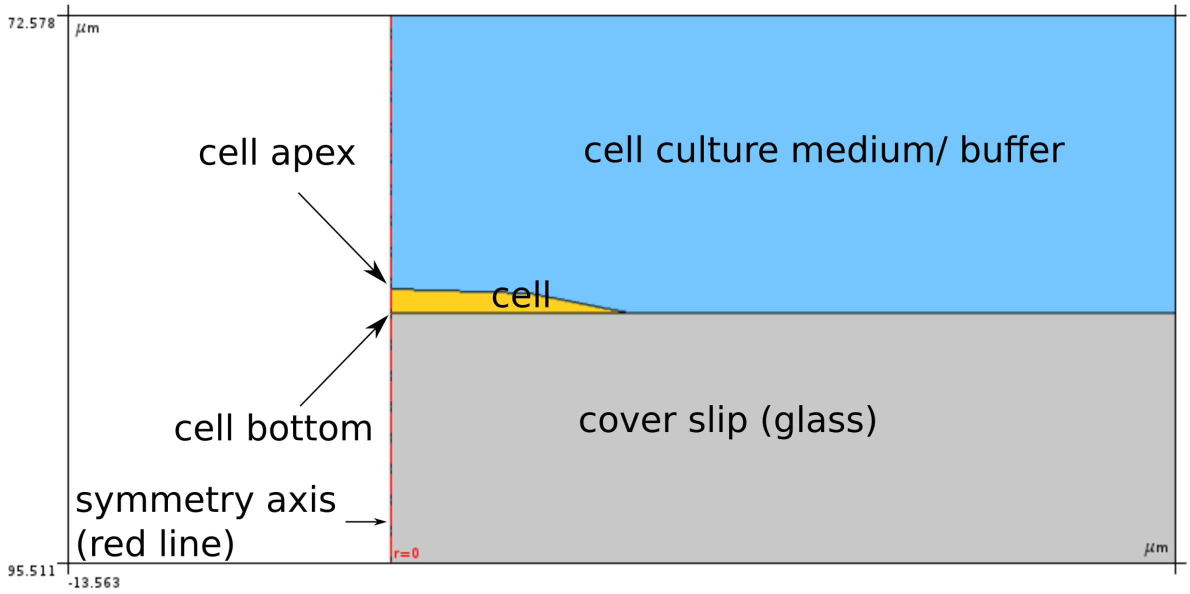

Figure S6. Zoom into the geometrical model. A cell adhered to the cover slip is shown. For the hydrogel case, a similar geometry was used, where the cell is placed as shown here on the top surface of the hydrogel instead of the cover slip.

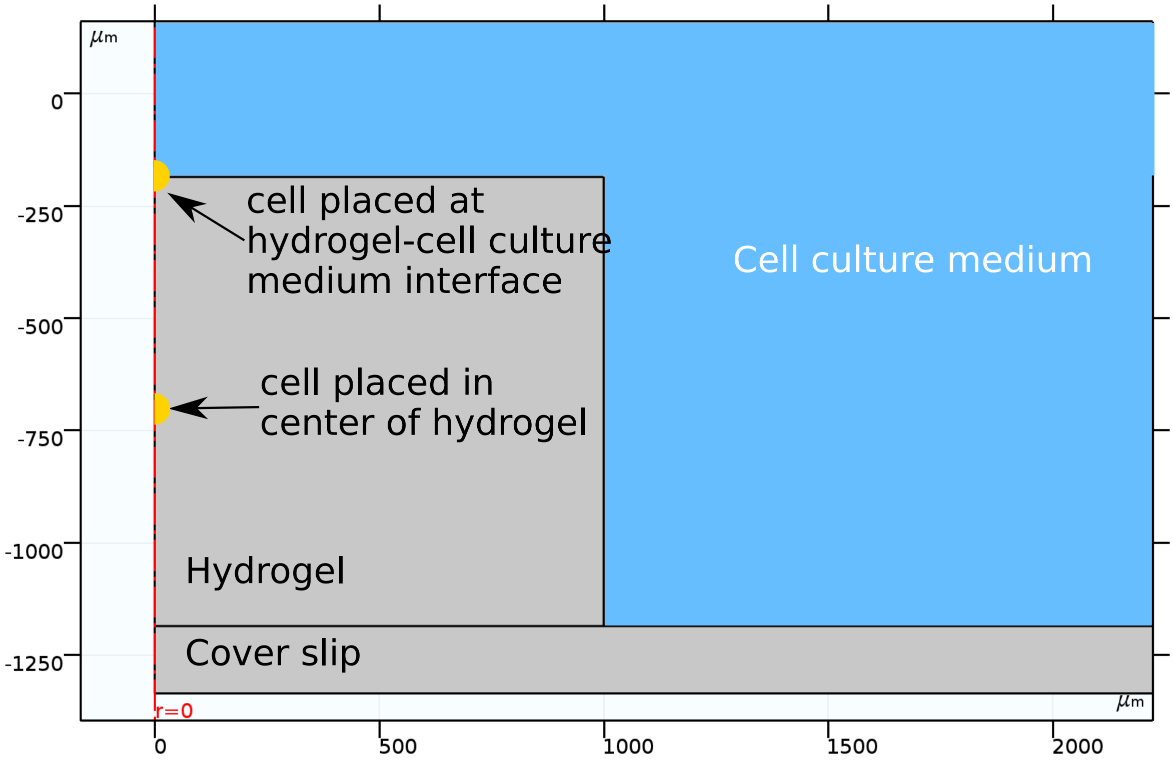

Figure S7. Zoom into the geometrical model with the hydrogel. The arrows demonstrate the two locations, where a single cell has been placed. The symmetry axis is again highlighted by the red line (compare also Fig. S6).

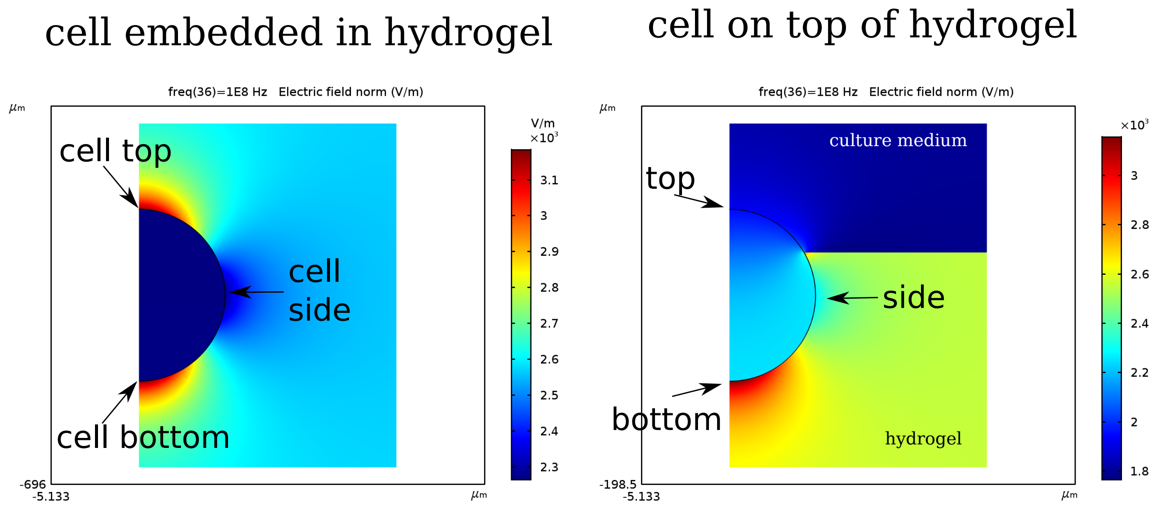

Figure S8. The electric field around the individual cells for two different configurations: (**a**) cell at the center of the hydrogel, (**b**) cell at the interface of hydrogel and cell culture medium.

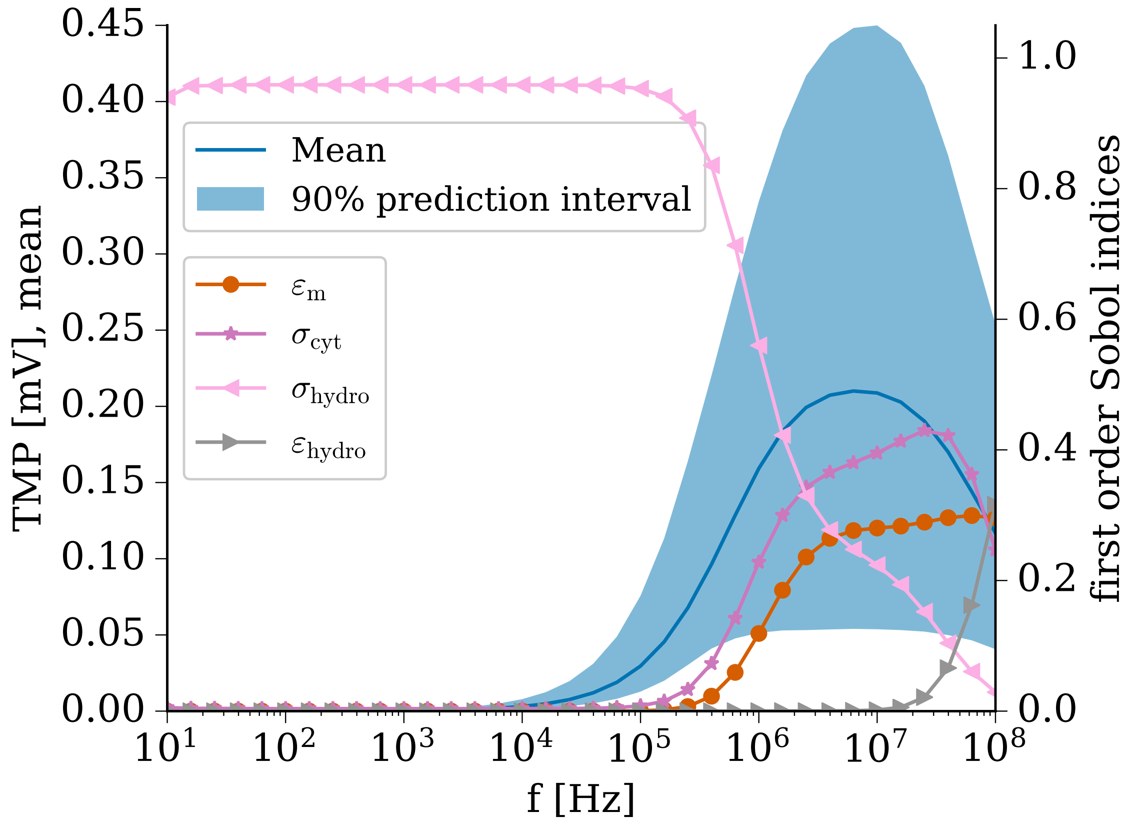

Figure S9. Mean value and 90% prediction interval for the TMP shown together with the Sobol indices for the scanned parameters of the configuration, where the cell is located at the center of the hydrogel. While the hydrogel conductivity σ_{hydro} has a large influence for frequencies below 1 MHz, at higher frequencies, cytoplasm conductivity σ_{cyt} and membrane permittivity ε_{m}) contribute substantially. Tested parameters whose Sobol index does not exceed 0.1 over the entire frequency range are not shown for the convenience of the reader.

4. Uncertainty Quantification result for the CM factor

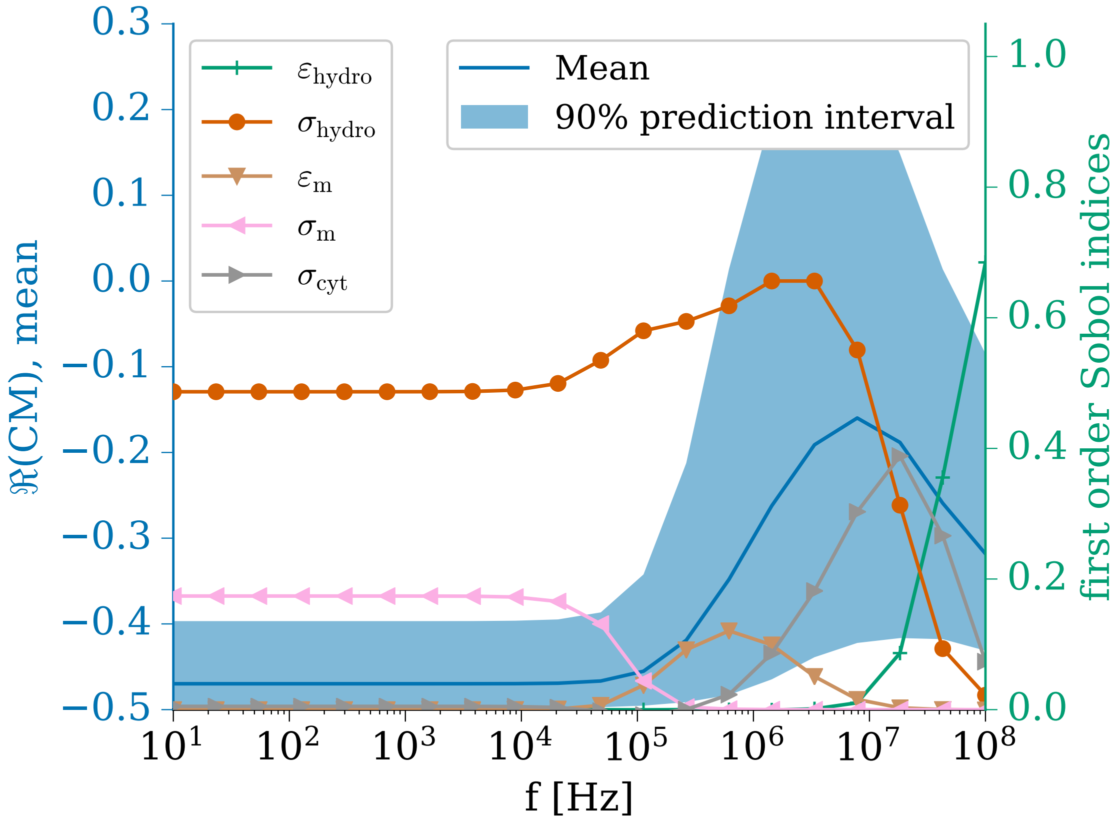

Figure S10. Mean value and 90% prediction interval for the real part of the CM factor, $\Re$(CM), shown together with the Sobol indices for the scanned parameters. The real part of the CM factor is a measure for the direction of the force on a spherical cell. While the membrane conductivity σ_{m} has a large influence for frequencies below 1 MHz, at higher frequencies, conductivity and permittivity of the hydrogel (σ_{buf} and ε_{buf}) as well as the conductivity of the cytoplasm σ_{cp} contribute substantially to $\Re$(CM). Tested parameters whose Sobol index does not exceed 0.1 over the entire frequency are not shown for the convenience of the reader.
