# Supplementary figures and images for: Numerical Simulations as Means for Tailoring Electrically Conductive Hydrogels towards Cartilage Tissue Engineering by Electrical Stimulation
Source: Molecules. 2020 Oct 16;25(20):4750. doi: 10.3390/molecules25204750 (PMC7587583; doi:10.3390/molecules25204750)

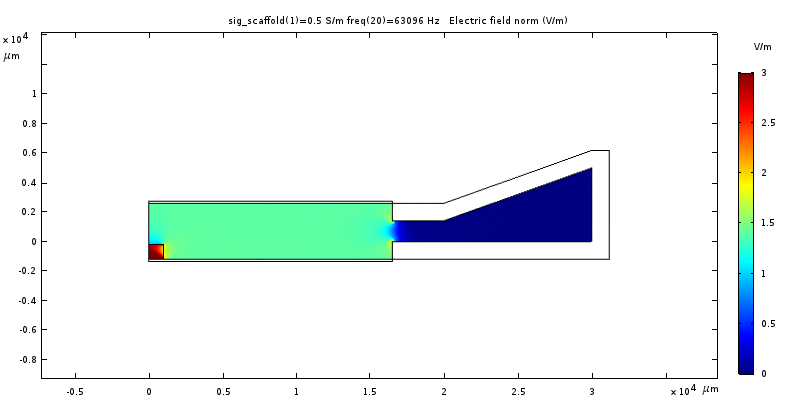

Supplement: Supplementary file 1 [file molecules-25-04750-s001.zip › SI/s11.gif]

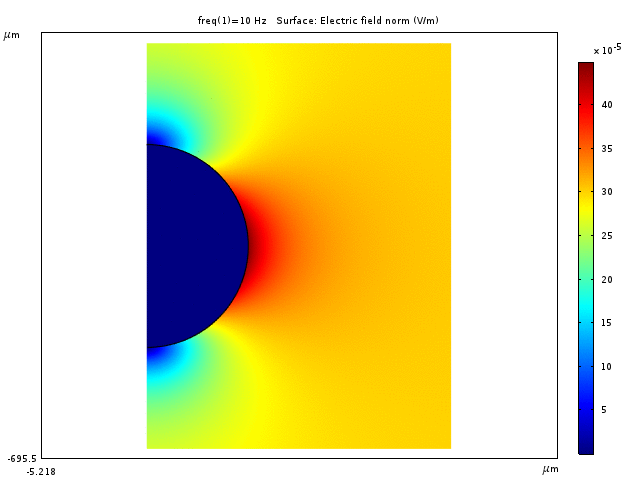

Supplement: Supplementary file 1 [file molecules-25-04750-s001.zip › SI/s12.gif]

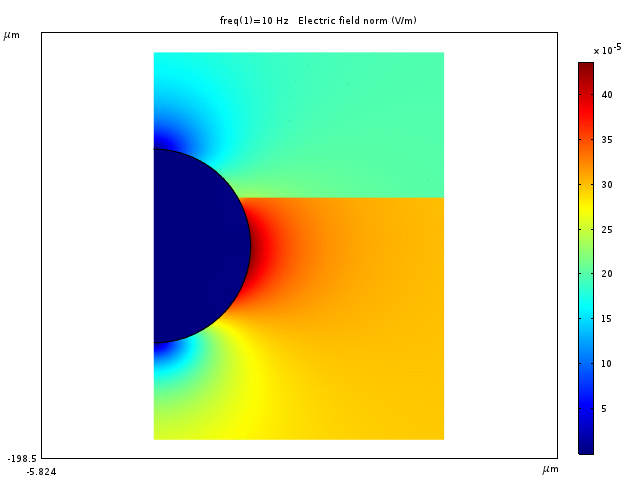

Supplement: Supplementary file 1 [file molecules-25-04750-s001.zip › SI/s13.gif]
